# Unexplored Nucleophilic Ring Opening of Aziridines †

**DOI:** 10.3390/molecules15129135

**Published:** 2010-12-10

**Authors:** Ana María Costero, Salvador Gil, Margarita Parra, Pablo Rodríguez

**Affiliations:** 1 Instituto de Reconocimiento Molecular y Desarrollo Tecnológico (IDM), Centro Mixto Universidad Politécnica de Valencia, Universidad de Valencia, 46100 Burjassot, Spain; 2 Department de Organic Chemistry, Universitat de València, Dr. Moliner 50, 46100 Burjassot, Spain; E-Mails: Salvador.gil@uv.es (S.G.); roapa@alumni.uv.es (P.R.); Ana.costero@uv.es (A.M.C.)

**Keywords:** enediolate, regioselectivity, diastereoselectivity, aziridines, γ-aminoacids

## Abstract

The reactivity of dianions of carboxylic acids towards aziridines has been studied. Although, a similar reactivity to that of enolates from ketones, esters or amides has been observed, the method directly yields γ-aminoacids in one step. The method is complementary of previous results of enenediolate reactivity with other electrophiles. A comparative study with the reactivity of this enediolates with epoxides is included.

## Introduction

Aziridines have been recognized as an attractive building block for the synthesis of a variety of nitrogen-containing biologically active compounds [1,2] Their high ring strain energy promotes high reactivity and many nucleophiles are able to cleave the ring [3,4]. The ring opening of aziridines is a widely investigated reaction and has been used to generate a large number of functionalized organic compounds that are not easily accessible by other means. However, the efficiency of the ring opening reactions of aziridines are heavily dependent upon the nature of the substituents on the three-member ring amines, the nucleophile and the reaction conditions employed [1,2,3,4,5,6,7].

The presence of electron-withdrawing substituents on the nitrogen atom activates the ring that then reacts easily with nucleophiles to form ring-opened products. In contrast, non-activated aziridines are relatively inert towards nucleophiles. Enolates derived from ketones, esters and amides have been used as effective nucleophiles to undergo addition to aziridines. The application of the enolate addition to aziridines has largely occurred in steroselective ring opening to form γ-amino carbonyl difunctionalized derivatives [3].

In order to extend our methodology using enediolates from carboxylic acids we wished to complete the study of nucleophilic ring opening with these nucleophiles, which permits direct acces to γ-aminoacids, as a full complement to the efficient synthesis of these compounds from enediolates of carboxylic acids and bromoacetonitrile, described previously by us [8,9].

## Results and Discussion

Carboxylic acids are synthetically useful building blocks because, after double deprotonation, they afford enediolates (or dienediolates when starting from α,β-unsaturated carboxylic acids) that react with various electrophiles under adequate conditions [10,11,12]. Lithium dialkylamides are commonly used as bases to generate the lithium dianions [10,11,12,13], due to their strength and low nucleophilicity, specially when derived from sterically hindered amines, combined with their solubility in non-polar solvents [13,14]. It is well established that, in these solvents, lithium enolates exist as complex ion pair aggregates, whose metal center may be coordinated to solvent molecules or other chelating ligands, such as the amines resulting from deprotonation of the acid by the lithium amide. The available data confirm the complexity present in those aggregated reactive species, whose reactivity and selectivity products can be influenced by many factors [10,11,12,13,14,15,16,17,18].

**Scheme 1:**
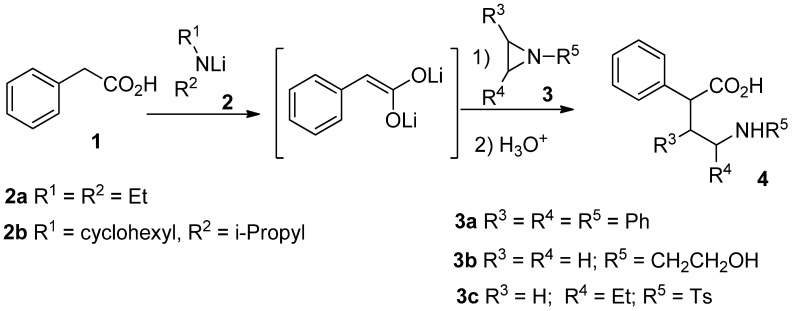
Reaction of aziridines with enediolate of phenylacetic acid.

We began describing the optimization of the addition reaction of enediolate of phenylacetic acid (**1**) with aziridines (Scheme 1). We have used in a first experience the commercially available aziridines **3a** and **3b** and lithium diethylamide as base to generate the dianion under standard condition [19] (Table 1, entries 1 and 2). In both reactions, only starting material was recovered. Although this was expected from the reactivity of non-activated aziridines with nucleophiles, it was worth testing as enediolates have shown a distinctive reactivity from other nucleophiles [9,10,11]. Thus, the more reactive 2-ethyl-1-tosylaziridine (**3c**), synthesized as described [20], was used in the rest of the experiments.

Two amines were tested as a base to generate the dienediolates, being cyclohexylisopropilamine **2b** the most efficient in these reactions. Previous studies lead us to develop sub-stoichiometric lithium amide conditions for the generation of dianions of carboxylic acids, which, in some cases improve the yield and selectivity of the reaction [19]. These conditions are especially adequate when lithium amide attacks faster the electrophile than the dianion. In this case, sub-stoichiometric amount of the amide did not led to any improvement (Table 1, entry 6). The last two entries in Table 1 reproduce the best conditions that we obtained in the addition of dianions of carboxylic acids to epoxides [21,22] by using LiCl as a disaggregating agent in the enolate solution (entry 8) or as a Lewis acid activating the epoxide (entry 9) where the dianion solution was added (inverse addition) to a mixture of the electrophile with LiCl in THF.

In spite of the slight increase of yield when compared to entry 7, use of the latter procedure was discarded as it is more complex. The optimized conditions for reaction with aziridine **3c**, namely lithium cyclohexylisopropylamide in equimolecular amount to generate the dianion and 1h reaction time at room temperature was extended to the rest of carboxylic acids (Table 2).

**Figure 1:**
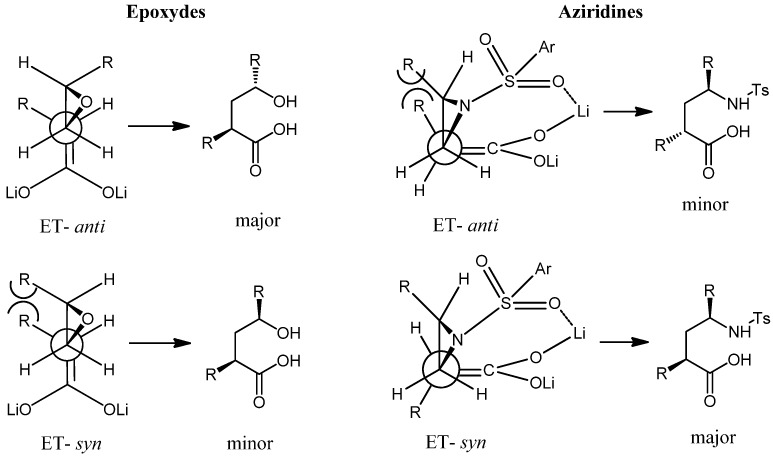
Transition States in the addition of enediolates to epoxydes and aziridines.

From non-conjugated carboxylic acids (*i.e*. **1**, **5** and **6)** γ-aminoacids were obtained straight away in moderate yield. As usual, the less hindered position of the aziridine was attacked to give compounds **4**, **13**, and **14**, with the *syn:anti* diasteroselectivity shown in Table 2. Despite the low diasteroselectivity, the *syn:anti* ratio was determined by NOESY studies. It is worth mention that the major *syn* selectivity contrast with the results obtained in the addition of carboxylic acid dianion to epoxides [22]. In cases like this, Taylor [23] consider the pre-transition state for S_N_2 type reaction, where the bulky groups appear to be too far away from each other to show a significant effect (Figure 1). In the reaction of the dianion with aziridine the sulfoniloxy group play an important role in the pre-transition state by means of their coordination with the lithium ions. This can overcome steric hindrance and change the diastereoselective ratio. Similar effect has been observed by us in the regioselective alkylation of dienediolates of carboxylic acids with tosylates [24].

On the other hand, we have extended this methodology to α,β-unsaturated carboxylic acids, whose double deprotonation lead to dienediolates that behave as ambident nucleophiles through their α or γ carbon atoms [10,11,12]. Although α attack predominates for irreversible reactions, strong deviations are observed in alkylation reactions [25,26]. Steric and electronic effects and the aggregation states in the middle of reaction determine the regioselectivity of the reaction with each electrophile.

Reactions of dimethylacrilic acid (**8**) and crotonic acid (**9**) with aziridines showed no regioselectivity, in adition products from crotonic acid (**9**) showed to be unstable and decomposed on purification. Only for tiglic acid (**7**) with a methyl group in the α position, is the γ-adduct the only regioisomer observed.

The diasteroselectivity of α−products from crotonic acid was not determined, but in the case of α-adducts **16**, the ratio was similar to that found for α-adducts from saturated acids, and as before, the corresponding γ-aminoacids were obtained. The method can be extended to *o*-methyl aromatic acids **10**, **11**, and **12** leading to aminoacids **20**, **21** and **22** in 40–60% yield.

## Conclusions

In summary, we have checked that the dianions of carboxylic acids react with aziridines in a similar way than enolates from ketones, esters or amides. In this case though, γ-aminoacids are directly obtained in one step. In those reactions leading to diastereosimers a moderate *syn* selectivity is observed. Thus, highly functionalized small molecules are obtained which are interesting building blocks to other transformations.

## Experimental 

### General

IR spectral data were obtained for liquid films between KBr discs, and the measurements were carried out by the SCSIE (Servei Central de Suport a la Investigació Experimental de la Universitat de Valencia) on a Matteson Satellite FTIR 3000 model Spectrophotometer. NMR spectra were recorded at 25 °C for solutions in the stated solvent on Bruker Avance 300 or 400 spectrometers. High resolution mass spectra were determined with a Fison VG Autospec spectrometer. Flash Column Silica Gel (230–400 mesh, Scharlau) was used for flash column chromatography, with hexane/ethyl acetate mixtures for elution. All reactions were carried out under argon atmospheres, in oven dried glassware, using standard conditions for exclusion of moisture. THF was freshly distilled from blue benzophenone ketyl and amines were distilled from CaH_2_ and stored over molecular sieves and kept under Ar. The BuLi used was a 1.6 M hexane solution. This solution’s concentration was periodically checked before use. The −78 °C reaction temperature was achieved by cooling with a CO_2_/acetone bath and 0 °C achieved by an ice/water bath. Organic extracts were dried over anhydrous MgSO_4_, and solutions were evaporated under reduced pressure with a rotatory evaporator and a bath set at 40 °C.

### General procedure for the reaction of lithium enediolates with N-tosylaziridine

*n*-BuLi (1.6 M in hexane, 3.1 mL, 5 mmol) was introduced into a previously purged reaction flask. The hexane was evaporated under vacuum and THF (2 mL), followed by cyclohexylisopropylamine (0.83 mL, 5 mmol) were added at -78 °C. The mixture was stirred for 15 min at 0 °C. The acid (2.25 mmol) in THF (2 mL) was added slowly at −78 °C and the mixture was kept at 0 °C for 30 min. *N*-tosyl-2-ethylaziridine (506 mg, 2.25 mmol) in THF (2 mL) was added slowly at −78 °C. The solution was stirred at room temperature for 1 h and quenched with H_2_O (15 mL). The reaction mixture was extracted with Et_2_O (3 × 15 mL). The aqueous phase at 0 °C, was acidified to pH 1 with conc. HCl and then extracted with EtOAc (3 × 15 mL) and the combined extracts were dried over anh. MgSO_4_. After evaporation of the solvent, the corresponding aminoacid was obtained.

*2-Phenyl-4-(4-methylphenylsulfonamido)hexanoic acid* (**4**). From phenylacetic acid (**1**, 306 mg); yield: 544 mg (67%); yellow oil; IR (KBr): υ = 3500–2700, 3258, 2974, 1710, 1600, 1496, 1367, 1164, 978, 712 cm^−1^; HRMS: m/z calcd. for C_19_H_23_NO_4_S [M^+^]: 361.1348; found: 361.1358. MS: m/z (%) = 361 [M^+^, 1%]; 343 [M^+^-H_2_O]; 332 [M^+^-CH_3_CH_2_, 34%]; 279 [C_16_H_23_O_4_^+^, 46%]; 212 [C_13_H_10_NO_2_^+^, 43%]. (2R*, 4R*) ^1^H-NMR (400 MHz, CDCl_3_): δ = 0.69 (t, *J* = 7.5 Hz, 3H, CH_3_CH_2_); 1.35 (m, 2H, CH_3_CH_2_); 1.92 (m, 1H, CHCHHCH); 2.23 (m, 1H, CHCHHCH); 2.41 (s, 3H, PhCH_3_); 3.17 (m, 1H, CHNH); 3.74 (t, *J* = 6.9 Hz, 1H, CHCOOH); 5.58 (d, *J* = 9.3 Hz, 1H, NH); 7.26 (m, 7H, CH_Ar_); 7.75 (d, *J* = 8.4 Hz, 2H, CH_Ar_) ppm; ^13^C-NMR (100 MHz, CDCl_3_): δ = 9.3 (CH_3_CH_2_); 21.5 (PhCH_3_); 28.2 (CH_3_CH_2_); 37.6 (CHCH_2_CH); 48.1 (CHCOOH); 53.7 (CHNH); 127.0 (CH_Ar_); 128.2 (CH_Ar_); 128.7 (CH_Ar_); 128.8 (CH_Ar_); 129.7 (CH_Ar_); 137.8 (C_Ar_); 138.4 (C_Ar_); 143.2 (C_Ar_); 179.2 (COOH) ppm. (2R*, 4S*) ^1^H-NMR (400 MHz, CDCl_3_): δ = 0.73 (t, *J* = 7.2 Hz, 3H, CH_3_CH_2_); 1.35 (m, 2H, CH_3_CH_2_); 1.70 (m, 1H, CHCHHCH); 2.23 (m, 1H, CHCHHCH); 2.38 (s, 3H, PhCH_3_); 3.18 (m, 1H, CHNH); 3.71 (t, *J* = 6.9 Hz, 1H, CHCOOH); 5.46 (d, *J* = 9.7 Hz, 1H, NH); 7.27 (m, 7H, CH_Ar_); 7.72 (d, *J* = 8.4 Hz, 2H, CH_Ar_) ppm; ^13^C-NMR (100 MHz, CDCl_3_): δ = 9.4 (CH_3_CH_2_); 21.0 (PhCH_3_); 27.9 (CH_3_CH_2_); 38.1 (CHCH_2_CH); 47.6 (CHCOOH); 53.5 (CHNH); 127.1 (CH_Ar_); 127.5 (CH_Ar_); 128.2 (CH_Ar_); 128.8 (CH_Ar_); 129.6 (CH_Ar_); 137.8 (C_Ar_); 138.5 (C_Ar_); 143.3 (C_Ar_); 178.6 (COOH) ppm.

*(E)-2-methyl-6-(4-methylphenylsulfonamido)oct-2-enoic acid* (**15**). From 2-methyl-2-butenoic acid (**1**, 225 mg); yield: 415 mg (68%); brown oil; IR (KBr): υ = 3400–2700, 2923, 2850, 1698, 1417, 1325, 1159, 1093, 815, 667 cm^−1^; HRMS: m/z calcd. for C_16_H_23_NO_4_S: 325,1348; found: 325.1422; MS: *m*/*z* (%):325 [M+, 97%]; 308 [M^+^-OH, 100%]; 212 [C_10_H_14_NO_2_S^+^, 23%]; 155 [C_7_H_7_O_2_S^+^, 48%]. ^1^H-NMR (300 MHz, CDCl_3_): δ = 0.75 (t, *J* = 7.3 Hz, 3H, CH_3_CH_2_); 1.30-2.00 (m, 4H, CH_2_CHCH_2_); 2.17 (s, 3H, CH_3_CCOOH); 2.32 (m, 2H, CHCH_2_CH_2_); 2.41 (s, 3H, PhCH_3_); 3.12 (m, 1H, CHNH); 6.96 (m, 1H, CH=CCOOH); 7.21 (d, *J* = 7.9 Hz, 2H, CH_Ar_); 7.73 (d, *J* = 8.1 Hz, 2H, CH_Ar_); ^13^C-NMR (75 MHz, CDCl_3_): δ = 9.81 (CH_3_CH_2_); 21.9 (PhCH_3_); 28.7 (CH_3_CCOOH); 36.6 (CH_3_CH_2_); 41.2 (=CHCH_2_CH_2_); 46.9 (=CHCH_2_CH_2_); 53.7 (CHNH); 119.2 (CH=CCOOH); 127.4 (CH_Ar_); 130.1 (CH_Ar_); 135.4 (C_Ar_); 138.7 (C_Ar_); 143.8 (CCOOH); 171.8 (COOH).

*Reaction with 3-methyl-2-butenoic acid:* From **8** (225 mg); yield: 366 mg (60%) as a 59:41 mixture of **16** and **17**

*4-(4-Methylphenylsulfonamido)-2-(prop-1-en-2-yl)hexanoic acid* (**16**): brown oil; IR (KBr): υ = 3300–2900, 3272, 2925, 1696, 1644, 1455, 1321, 1158, 1092, 814, 666 cm^−1^; HRMS: m/z calcd. for C_16_H_23_NO_4_S: 325.1348; found: 325.1335; MS: m/z (%) = 325 [M+, 1%]; 308 [M+-OH, 100%]; 212 [C_10_H_14_NO_2_S^+^, 19%]; 155 [C_7_H_7_O_2_S^+^, 25%]. ^1^H-NMR (400 MHz, CDCl_3_): δ = 0.74 (t, *J* = 7.4 Hz, 3H, CH_3_CH_2_); 1.27 (m, 4H, CH_2_CHCH_2_); 1.85 (s, 3H,); 2.43 (s, 3H, PhCH_3_); 3.15 (m, 2H, CHNH, CHCOOH); 4.83 (s, 1H, CHH=CCH_3_), 4.93 (s, 1H, CHH=CCH_3_); 7.34(d, *J* = 8.0 Hz, 2H, CH_Ar_); 7.81 (d, *J* = 8.1 Hz, 2H, CH_Ar_) ppm; ^13^C-NMR (100 MHz, CDCl_3_): δ = 9.3 (CH_3_CH_2_); 20.3 (CH_3_C=CH_2_); 25.2 (PhCH_3_); 27.7 (CH_3_CH_2_); 34.4 (CHCH_2_CH); 50.7 (CHNH); 54.0 (CHCOOH); 114.9 (CH_3_C=CH_2_); 127.1 (CH_Ar_); 129.6 (CH_Ar_); 138.5 (C_Ar_); 143.2 (C_Ar_); 163.3 (CH_3_C=CH_2_); 178.8 (COOH) ppm.

*(E)-3-Methyl-6-(4-methylphenylsulfonamido)oct-2-enoic acid* (**17**): brown oil; ^1^H-NMR (400 MHz, CDCl_3_): δ = 0.76 (t, *J* = 7.5 Hz, 3H, CH_3_CH_2_); 1.43 (m, 4H, CH_3_CH_2_, CCH_2_); 2.33 (dt, *J_1_* = 5.1 Hz, *J_2_* = 7.4 Hz, 1H, CCH_2_CHH); 2.42 (s, 3H, PhCH_3_); 2.52 (dt, *J_1_* = 5.2 Hz, *J_2_* = 7.4 Hz, 1H, CCH_2_CHH); 3.19 (m, 1H, CHNH); 5.66 (s, 1H, CHCOOH); 7.29 (d, *J* = 7.8 Hz, 2H, CH_Ar_); 7.78 (d, *J* = 8.3 Hz, 2H, CH_Ar_) ppm; ^13^C-NMR (100 MHz, CDCl_3_): δ = 9.8 (CH_3_CH_2_); 21.5 (CH_3_CCH_2_); 28.4 (PhCH_3_); 29.5 (CH_3_CH_2_); 32.8 (CHCH_2_CH_2_); 34.4 (CHCH_2_CH_2_); 55.5 (CHNH); 115.7 (CHCOOH); 127.0 (CH_Ar_); 129.6 (CH_Ar_); 138.3 (C_Ar_); 143.2 (C_Ar_); 163.3 (C=CHCOOH); 171.3 (COOH) ppm.

*4-(4-Methylphenylsulfonamido)-2-propylhexanoic acid* (**13**). From pentenoic acid (**5**, 230 mg); yield: 198 mg (31%); yellow oil; IR (KBr): υ = 3500–2700, 3235, 2917, 1712, 1591, 1465, 1382, 1131, 1021, 732 cm^−1^; HRMS: m/z calcd. for C_16_H_25_NO_4_S: 327,1504, found: 327.1979; MS: *m*/*z* (%): 327 [M^+^, 16%]; 311 [C_16_H_25_NO_3_S^+^, 20%]; 310 [M^+^-OH, 100%]; (2R*, 4R*) ^1^H-NMR (300 MHz, CDCl_3_): δ = 0.77 (t, *J* = 7.5 Hz, 3H, CH_3_CH_2_CH_2_); 0.96 (t, *J* = 7.6 Hz, 3H, CH_3_CH_2_CH); 1.30-2.00 (m, 9H, CH_3_CH_2_CH_2_CHCH_2_CHCH_2_); 2.51 (s, 3H, PhCH_3_); 3.31 (bs, 1H, CHNH); 7.37 (d, *J* = 8.1 Hz, 2H, CH_Ar_); 8.20 (d, *J* = 8.4 Hz, 2H, CH_Ar_) ppm; ^13^C-NMR (75 MHz, CDCl_3_): δ = 9.0 (CH_3_CH_2_CH); 14.1 (CH_3_CH_2_CH_2_); 21.9 (CH_3_CH_2_CH_2_); 27.6 (CH_3_CH_2_CH); 30.3 (CH_3_CH_2_CH_2_); 35.1 (CHCH_2_CH); 41.5 (CHCOOH); 54.3 (CHNH); 127.4 (CH_Ar_); 129.8 (CH_Ar_); 138.8 (C_Ar_); 143.7 (C_Ar_); 181.6 (COOH) ppm. (2R*, 4S*) ^1^H-NMR (300 MHz, CDCl_3_): δ = 0.79 (t, *J* = 7.5 Hz, 3H, CH_3_CH_2_CH_2_); 0.98 (t, *J* = 7.7 Hz, 3H, CH_3_CH_2_CH); 1.30-2.00 (m, 9H, CH_3_CH_2_CH_2_CHCH_2_CHCH_2_); 2.50 (s, 3H, PhCH_3_); 3.31 (bs, 1H, CHNH); 7.38 (d, *J* = 8.0 Hz, 2H, CH_Ar_); 7.86 (d, *J* = 8.3 Hz, 2H, CH_Ar_) ppm; ^13^C-NMR (75 MHz, CDCl_3_): δ = 9.1 (CH_3_CH_2_CH); 13.8 (CH_3_CH_2_CH_2_); 22.0 (CH_3_CH_2_CH_2_); 28.3 (CH_3_CH_2_CH); 30.3 (CH_3_CH_2_CH_2_); 35.0 (CHCH_2_CH); 41.4 (CHCOOH); 54.6 (CHNH); 127.5 (CH_Ar_); 130.0 (CH_Ar_); 138.3 (C_Ar_); 143.6 (C_Ar_); 182.2 (COOH) ppm.

*2-Benzyl-4-(4-methylphenylsulfonamido)hexanoic acid* (**14**). From 3-phenylpropinoic acid (**6**, 338 mg); yield: 498 mg (59%); yellow oil; IR (KBr): υ = 3500–2700, 3270, 2930, 1708, 1599, 1495, 1322, 1159, 1092, 700 cm^−1^; HRMS: m/z calcd. for C_20_H_25_NO_4_S: 375.1504; found: 375.1525; MS: *m*/*z* (%): 375 [M^+^, 1%]; 357 [M^+^-H_2_O]; 293 [C_20_H_23_NO^+^, 22%]; 155 [C_7_H_7_O_2_S^+^, 32%]; 91 [C_7_H_7_^+^, 100%]; (2R*, 4R*) ^1^H-NMR (300 MHz, CDCl_3_): δ = 0.73 (t, *J* = 7.2 Hz, 3H, CH_3_CH_2_); 1.37 (m, 2H, CH_3_CH_2_); 1.42 (m, 1H, CHCHHCH); 1.92 (m, 1H, CHCHHCH); 2.50 (s, 3H, PhCH_3_); 2.84 (m, 2H, CHCOOH, PhCHH); 3.30 (m, 1H, PhCHH); 3.32 (bs, 1H, CHNH); 5.52 (d, *J* = 9.0 Hz, 1H, NH); 7.34 (m, 5H, CH_Ar_); 7.35 (d, *J* = 7.8 Hz, 2H, CH_Ar_); 7.83 (d, *J* = 8.1 Hz, 2H, CH_Ar_) ppm; ^13^C-NMR (75 MHz, CDCl_3_): δ = 9.7 (CH_3_CH_2_); 22.1 (PhCH_3_); 28.4 (CH_3_CH_2_); 36.2 (CHCH_2_CH); 38.6 (PhCH_2_); 44.6 (CHCOOH); 54.4 (CHNH); 126.9 (CH_Ar_); 127.4 (CH_Ar_); 128.7 (CH_A_r); 129.0 (CH_Ar_); 129.4 (CH_Ar_); 138.6 (C_Ar_); 140.5 (C_Ar_); 143.7 (C_Ar_); 181.1 (COOH) ppm. (2R*, 4S*) ^1^H-NMR (300 MHz, CDCl_3_): δ = 0.73 (t, *J* = 7.2 Hz, 3H, CH_3_CH_2_); 1.37 (m, 2H, CH_3_CH_2_); 1.42 (m, 1H, CHCHHCH); 1.96 (m, 1H, CHCHHCH); 2.50 (s, 3H, PhCH_3_); 2.84 (m, 2H, CHCOOH, PhCHH); 3.30 (m, 1H, PhCHH); 3.32 (bs, 1H, CHNH); 5.23 (d, *J* = 9.0 Hz, 1H, NH); 7.34 (m, 5H, CH_Ar_); 7.35 (d, *J* = 7.8 Hz, 2H, CH_Ar_); 7.84 (d, *J* = 8.1 Hz, 2H, CH_Ar_) ppm; ^13^C-NMR (75 MHz, CDCl_3_): δ = 10.1 (CH_3_CH_2_); 22.0 (PhCH_3_); 28.2 (CH_3_CH_2_); 36.0 (CHCH_2_CH); 38.4 (PhCH_2_); 43.4 (CHCOOH); 54.2 (CHNH); 126.8 (CH_Ar_); 127.4 (CH_Ar_); 128.6 (CH_A_r); 128.9 (CH_Ar_); 129.4 (CH_Ar_); 138.3 (C_Ar_); 140.5 (C_Ar_); 143.7 (C_Ar_); 179.2 (COOH) ppm.

*2-(3-(4-Methylphenylsulfonamido)pentyl)benzoic acid* (**20**). From 2-methylbenzoic acid (**10**, 306 mg); yield: 325 mg (40%); yellow oil; IR (KBr): υ = 3300–2900, 3272, 2926, 1696, 1600, 1455, 1321, 1158, 1093, 666 cm^−1^; HRMS: m/z calcd. for C_19_H_23_NO_4_S: 361.1348; found: 361.1443; MS: *m*/*z* (%): 361 [M^+^, 0.1%]; 343 [M^+^-H_2_O, 1%]; 332 [M^+^-CH_3_CH_2_, 30%]; 314 [C_17_H_16_NOS^+^, 63%]; 212 [C_13_H_10_NO_2_^+^, 100%]; 155 [C_7_H_7_O_2_S^+^, 85%]; 91 [C_7_H_7_^+^, 93%]. ^1^H-NMR (300 MHz, CDCl_3_): δ = 0.91 (t, *J* = 7.5 Hz, 3H, CH_3_CH_2_); 1.59 (m, 2H, CH_3_CH_2_); 1.77 (m, 2H, PhCH_2_CH_2_); 2.29 (s, 3H, PhCH_3_); 2.81 (dt, *J_1_* = 10.8, *J_2_* = 5.0, 1H, PhCHH); 3.06 (dt, *J1* = 10.6, *J2* = 5.3 Hz, 1H, PhCHH); 3.32 (m, 1H, CHNH); 5.64 (d, *J* = 7.5 Hz, 1H, NH); 7.37 (m, 4H, CH_Ar_); 7.55 (t, *J* = 6.0 Hz, 1H, CH_Ar_); 7.91 (d, *J* = 10.1 Hz, 1H, CH_Ar_); 8.16 (d, *J* = 6.6 Hz, 1H, CH_Ar_) ppm; ^13^C-NMR (75 MHz, CDCl_3_): δ = 10.2 (CH_3_CH_2_); 21.9 (PhCH_3_); 28.6 (CH_3_CH_2_); 30.9 (PhCH_2_); 36.9 (PhCH_2_CH_2_); 55.9 (CHNH); 126.6 (CH_Ar_); 127.5 (CH_Ar_); 129.9 (CH_Ar_); 130.1 (CH_Ar_); 131.6 (CH_Ar_); 132.4 (CH_Ar_); 133.4 (C_Ar_); 136.7 (C_Ar_); 143.4 (C_Ar_); 145.3 (C_Ar_); 173.2 (COOH) ppm.

*5-Methyl-2-(3-(4-methylphenylsulfonamido)pentyl)furan-3-carboxylic acid* (**21**). From 2,5-dimethyl-3-furanoic acid (**11**, 315 mg); yield: 435 mg (53%); brown oil; IR (KBr): υ = 3300–2900, 3271, 2927, 1682, 1583, 1435, 1323, 1235, 1159, 815 cm^−1^; HRMS: m/z calcd. for C_18_H_23_NO_5_S: 365,1297; found: 365.1292; MS: *m*/*z* (%): 365 [M^+^, 0.2%]; 347 [M^+^-H_2_O]; 212 [C_10_H_14_NO_2_S^+^, 49%]; 155 [C_7_H_7_O_2_S^+^, 50%]; 91 [C_7_H_7_^+^, 100%]; ^1^H-NMR (300 MHz, CDCl_3_): δ = 0.79 (t, *J* = 7.2 Hz, 3H, CH_3_CH_2_); 1.37 (m, 2H, CH_3_CH_2_); 1.64 (m, 2H, CHCH_2_CH_2_); 2.21 (s, 3H, CH_3_C-O); 2.51 (s, 3H, PhCH_3_); 2.70 (m, 1H, CHCH_2_CHH); 2.84 (m, 1H, CHCH_2_CHH); 3.15 (m, 1H, CHNH); 5.03 (d, *J* = 8.4 Hz, 1H, NH); 6.18 (s, 1H, CHCCOOH); 7.22 (d, *J* = 8.1 Hz, 2H, CH_Ar_); 7.70 (d, *J* = 8.1 Hz, 2H, CH_Ar_) ppm; ^13^C-NMR (75 MHz, CDCl_3_): δ = 10.1 (CH_3_CH_2_); 13.6 (CH_3_C-O); 21.9 (CCH_2_); 24.1 (PhCH_3_); 28.1 (CH_3_CH_2_); 33.0 (CCH_2_CH_2_); 55.4 (CHNH); 106.6 (CHCOOH); 113.6 (CCOOH); 127.4 (CH_Ar_); 130.1 (CH_Ar_); 138.6 (C_Ar_); 143.5 (C_Ar_); 151.0 (CH_3_C); 162.7 (CCH_2_); 169.8 (COOH) ppm.

*3-(3-(4-Methylphenylsulfonamido)pentyl)thiophene-2-carboxylic acid* (**22**). From 3-methyl-2-tiophenoic acid (**12**, 320 mg); yield: 496 mg (60%); brown oil; IR (KBr): υ = 3400–2600, 2967, 2639, 1671, 1598, 1537, 1428, 1303, 1158, 1093 cm^−1^; HRMS: m/z calcd. for C_17_H_21_NO_4_S_2_: 367,0912; found: 367.0912; MS: *m*/*z* (%): 367 [M^+^, 1%]; 338 [M^+^-CH_3_CH_2_, 27%]; 320 [C_15_H_14_NO_3_S_2_^+^, 36%]; 212 [C_10_H_14_NO_2_S^+^, 100%]; 155 [C_7_H_7_O_2_S^+^, 77%]; 91 [C_7_H_7_^+^, 78%]; ^1^H-NMR (400 MHz, CDCl_3_): δ = 0.78 (t, *J* = 7.4 Hz, 3H, CH_3_CH_2_); 1.48 (m, 2H, CH_3_CH_2_); 1.71 (m, 2H, CH_2_CH_2_CH); 2.40 (s, 3H, PhCH_3_); 2.84 (m, 1H, CHHCH_2_CH); 2.95 (m, 1H, CHHCH_2_CH); 3.20 (m, 1H, CH_2_CH); 6.89 (d, *J* = 5.0 Hz, 1H, SCH=CH); 7.27 (d, *J* = 8.0 Hz, 2H, CH_Ar_); 7.47 (d, *J* = 5.0 Hz, 1H; SCH=CH); 7.78 (d, *J* = 8.3 Hz, 2H, CH_Ar_) ppm; ^13^C-NMR (100 MHz, CDCl_3_): δ = 9.5 (CH_3_CH_2_); 21.5 (PhCH_3_); 25.6 (CH_2_CH_2_CH); 27.5 (CH_3_CH_2_); 31.5 (CH_2_CH_2_CH); 34.5 (CH_2_CH); 127.0 (CH_Ar_); 129.7 (CH_Ar_); 131.7 (SCH=CH); 132.1 (SCH=CH); 138.2 (C_Ar_); 143.2 (C_Ar_); 147.9 (CCOOH); 151.8 (CCH_2_); 167.6 (COOH) ppm.

## Figures and Tables

**Table 1:** Optimization of the reaction conditions of enediolate of phenylacetic acid with aziridines.

Entry	Aziridine	Amine	Time at r.t.	Yield (%)
1	**3a**	**2a**	1h	0
2	**3b**	**2a**	1h	0
3	**3c**	**2a**	1h	50
4	**3c**	**2a**	3h	44
5	**3c**	**2a**	24h	35
6	**3c**	2a*	1h	46
7	**3c**	**2b**	1h	67
8	**3c**	**2b**	1h	58**
9	**3c**	**2b**	1h	71**

* 0.5 eq. of amine; ** Using LiCl as additive in normal (entry 8) or inverse addition (entry 9).

**Table 2:** Addition of dianions of carboxylic acids to aziridine **3c**.

Entry	Acid	Product	Yield (%)	γ : α	*Syn : Anti*
1	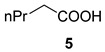	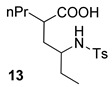	31		66 : 33
2	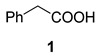	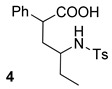	67		64 :36
3	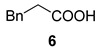	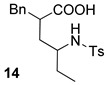	59		67 : 33
4	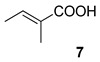	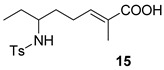	68	100 : 0	
5	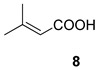	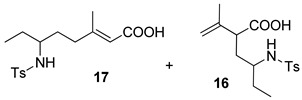	60	41 : 59	69 : 31
6	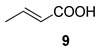	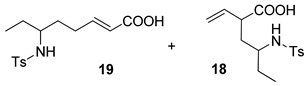	25	50 : 50	
7	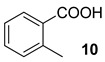	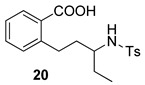	40		
8	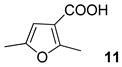	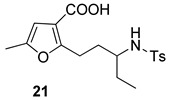	53		
9	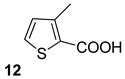	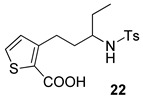	60		
